# Supplementary material for: Comparison of Lung Ultrasound versus Chest X-ray for Detection of Pulmonary Infiltrates in COVID-19
Source: Diagnostics (Basel). 2021 Feb 22;11(2):373. doi: 10.3390/diagnostics11020373 (PMC7926899; doi:10.3390/diagnostics11020373)
Supplement: Supplementary file 1 [file diagnostics-11-00373-s001.zip › diagnostics-1114995-supplementary.pdf]

diagnostics

MDPI

Supplementary Tables and Figures

Table 1. Number of lobes with pulmonary infiltrates detected by chest radiograph stratified by the suspected and confirmed groups of patients with COVID-19.

Number of Lobes	Suspected $n = 53$ (%)	Confirmed $n = 43$ (%)	Total $n = 96$ (%)	p value
**Right Lung**				**<0.001**
0	31 (58.5)	10 (23.3)	41 (42.7)	
1	16 (30.2)	8 (18.6)	24 (25.0)	
2	4 (7.5)	20 (46.5)	24 (25.0)	
3	2 (3.8)	5 (11.6)	7 (7.3)	
**Left Lung**				**<0.001**
0	34 (64.2)	6 (14.0)	40 (41.7)	
1	15 (28.3)	18 (41.9)	33 (34.4)	
2	4 (7.5)	19 (44.2)	23 (24.0)	
**Both Lungs**				**<0.001**
0	28 (52.8)	4 (9.3)	32 (33.3)	
1	8 (15.1)	5 (11.6)	13 (13.5)	
2	11 (20.8)	7 (16.3)	18 (18.8)	
3	2 (3.8)	12 (27.9)	14 (14.6)	
4	3 (5.7)	11 (25.6)	14 (14.6)	
5	1 (1.9)	4 (9.3)	5 (5.2)	

Table 2. Number of lobes with pulmonary infiltrates detected by lung ultrasound stratified by the suspected and confirmed groups of patients with COVID-19.

Number of Lobes	Suspected $n = 53$ (%)	Confirmed $n = 43$ (%)	Total $n = 96$ (%)	p value
**Right Lung**				**0.013**
0	18 (34.0)	4 (9.3)	22 (22.9)	
1	10 (18.9)	9 (20.9)	19 (19.8)	
2	15 (28.3)	15 (34.9)	30 (31.3)	
3	6 (11.3)	3 (7.0)	9 (9.4)	
4	4 (7.5)	8 (18.6)	12 (12.5)	
5	0	4 (9.3)	4 (4.2)	
**Left Lung**				**<0.001**
0	27 (50.9)	5 (11.6)	32 (33.3)	
1	13 (24.5)	8 (18.6)	21 (21.9)	
2	6 (11.3)	16 (37.2)	22 (22.9)	
3	4 (7.5)	8 (18.6)	12 (12.5)	
4	3 (5.7)	4 (9.3)	7 (7.3)	
5	0	2 (4.7)	2 (2.1)	
**Both Lungs**				**0.011**
0	16 (30.2)	2 (4.7)	18 (18.8)	
1	6 (11.3)	3 (7.0)	9 (9.4)	
2	10 (18.9)	2 (4.7)	12 (12.5)	
3	5 (9.4)	9 (20.9)	14 (14.6)	
4	8 (15.1)	10 (23.3)	18 (18.8)	
5	2 (3.8)	4 (9.3)	6 (6.3)	

6	3 (5.7)	5 (11.6)	8 (8.3)
7	2 (3.8)	2 (4.7)	4 (4.2)
8	1 (1.9)	3 (7.0)	30 (31.3)
9	0	2 (4.7)	2 (2.1)
10	0	2 (2.3)	1 (1.0)

Table 3. Detection of pulmonary infiltrates (none, unilateral, or bilateral) by lung ultrasound versus disposition to discharge home in all, suspected, and confirmed patients with COVID-19.

**Lung Ultrasound**				***p* value**
**All**	**Home** ***n* = 58 (%)**	**Admitted** ***n* = 38 (%)**	**Total** ***n* = 96 (%)**	0.001
None	16 (27.6)	2 (5.3)	18 (18.0)	
Unilateral	14 (24.1)	4 (10.5)	18 (18.8)	
Bilateral	28 (48.3)	32 (84.2)	60 (62.5)	
**Suspected**	**Home** ***n* = 50 (%)**	**Admitted** ***n* = 3 (%)**	**Total** ***n* = 53 (%)**	0.146
None	16 (32.0)	0 (0.0)	16 (30.2)	
Unilateral	13 (26.0)	0 (0.0)	13 (24.5)	
Bilateral	21 (42.0)	3 (100.0)	24 (45.3)	
**Confirmed**	**Home** ***n* = 8 (%)**	**Admitted** ***n* = 35 (%)**	**Total** ***n* = 43 (%)**	0.786
None	0 (0.0)	2 (5.7)	2 (4.7)	
Unilateral	1 (12.5)	4 (11.4)	5 (11.6)	
Bilateral	7 (87.5)	29 (32.9)	36 (83.7)	

Table 4. Detection of pulmonary infiltrates (none, unilateral, or bilateral) by chest X-ray versus disposition to discharge home in all, suspected, and confirmed patients with COVID-19.

**Chest X-Ray**				***p* value**
**All**	**Home** ***n* = 58 (%)**	**Admitted** ***n* = 38 (%)**	**Total** ***n* = 96 (%)**	<0.001
None	35 (60.3)	1 (2.6)	36 (37.5)	
Unilateral	9 (15.5)	5 (13.2)	14 (14.6)	
Bilateral	14 (24.1)	32 (84.2)	46 (47.9)	
**Suspected**	**Home** ***n* = 50 (%)**	**Admitted** ***n* = 3 (%)**	**Total** ***n* = 53 (%)**	0.161
None	31 (62.0)	1 (33.3)	32 (60.4)	
Unilateral	9 (18.0)	0 (0.0)	9 (17.0)	
Bilateral	10 (20.0)	2 (66.7)	12 (22.6)	
**Confirmed**	**Home** ***n* = 8 (%)**	**Admitted** ***n* = 35 (%)**	**Total** ***n* = 43 (%)**	–
None	4 (50.0)	0 (0.0)	4 (9.3)	
Unilateral	0 (0.0)	5 (14.3)	5 (11.6)	
Bilateral	4 (50.0)	30 (85.7)	34 (79.1)	

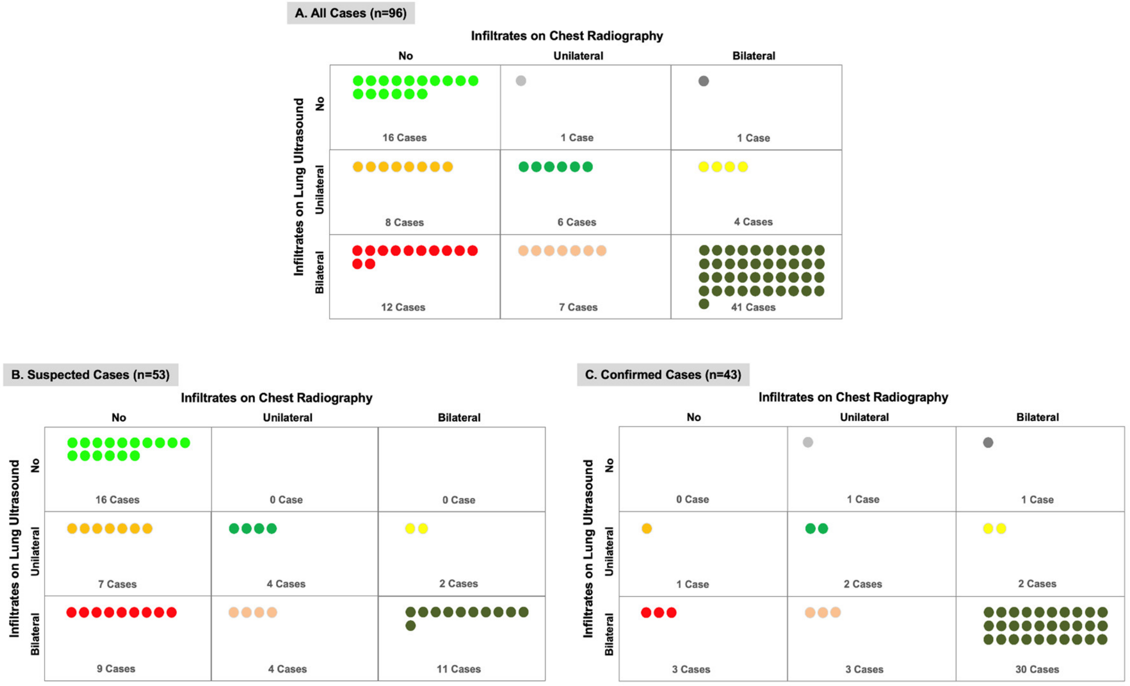

Figure 1. Comparison of Normal, Unilateral and Bilateral Infiltrates on Chest X-ray and Lung Ultrasound. The agreement between chest X-ray and lung ultrasound is shown for (**A**) all cases, (**B**) suspected COVID-19 cases, and (**C**) confirmed COVID-19 cases. Lung ultrasound detected bilateral pulmonary infiltrates in a substantial proportion of subjects with either a normal or unilateral infiltrates on chest X-ray.

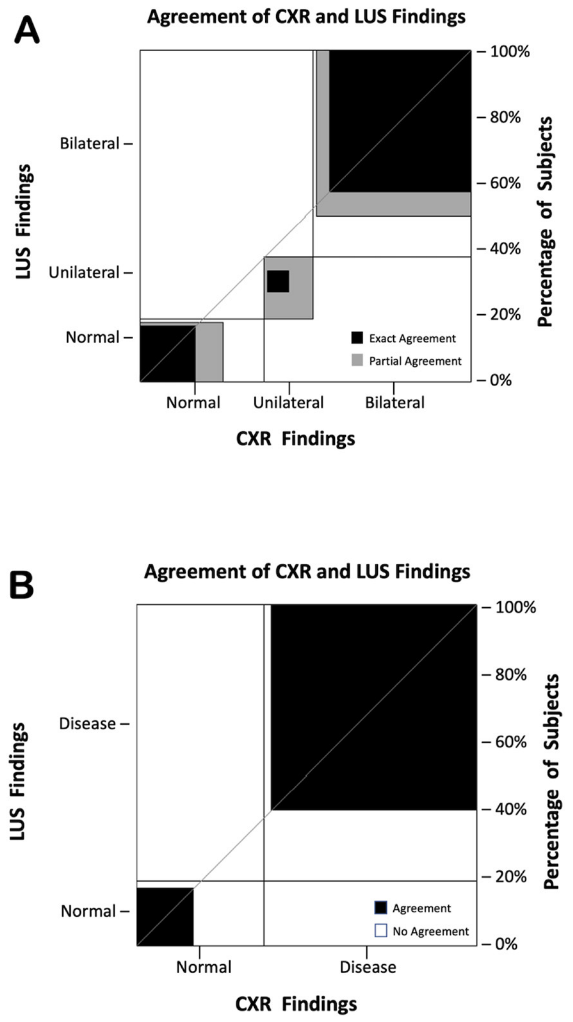

Figure 2. Correlation of Lung Ultrasound and Chest X-ray in COVID-19. (**A**) This plot shows the correlation of findings for normal, unilateral, or bilateral disease. (**B**) This plot shows the correlations between normal and any disease. The darkest areas indicate exact agreement between LUS and CXR, lightest areas indicate partial or no agreement between LUS and CXR. The 45-degree line above the intersection of the middle rectangles indicates that LUS (plotted vertically) detects more disease than does CXR (plotted horizontally).
